# Passive dehydration alters raw bioimpedance parameters: generic vs. individualized phase angle and estimated characteristic frequency

**DOI:** 10.1080/15502783.2025.2550182

**Published:** 2025-08-26

**Authors:** David H. Fukuda, Modesto A. Lebron, Kworweinski Lafontant, Gavin N. Van Staden, Adam J. Wells, Jeffrey R. Stout

**Affiliations:** a University of Central Florida, Institute of Exercise Physiology and Rehabilitation Science, Orlando, FL; b University of Central Florida, College of Medicine, Orlando, FL

**Keywords:** Match duration, beach volleyball, performance metrics, game evolution, athletic performance

## Abstract

**Background:**

Bioelectrical impedance analysis (BIA) is commonly used to determine body composition, including estimates of total body water (TBW), intracellular water (ICW), and extracellular water (ECW). Evaluation of raw BIA data, i.e. resistance (R; as cellular hydration), reactance (Xc; as cellular integrity), and phase angle (PhA; as cellular health), has become more relevant when estimation equations may not be applicable, such as hypohydration in sport, occupational, or clinical settings. Bioimpedance values are typically determined at a generic characteristic frequency (Fc) of 50 kHz, which is intended to differentiate the ability of currents to penetrate cell membranes. Multi-frequency BIA allows for the estimation of Fc and its associated PhA at maximum reactance (PhA@Fc), which likely vary from assumed values on an individual basis or following certain physiological changes. Therefore, the purpose of this study was to examine these BIA parameters at rest and following passive dehydration.

**Methods:**

Fifteen healthy adults (11 males: 22.5 ± 2.7 yrs, 175.5 ± 6.5 cm, 76.4 ± 9.8 kg; 4 females: 21.0 ± 2.6 yrs, 163.1 ± 2.8 cm, 54.8 ± 4.3 kg) completed a dehydration trial in a portable infrared sauna. After a standardized warm-up, participants alternated up to 20 min of sauna exposures (~66°C) with 5 min rest in a thermoneutral setting (~23°C) until reaching a predetermined body mass loss (males = 2.8 ± 0.3%; females = 2.4 ± 0.3%). Outcome measures were collected using multi-frequency BIA device with touch-type electrodes at rest and following dehydration. PhA@Fc was derived using Cole–Cole modeling with 5-, 50-, and 250 kHz BIA data, and Fc was estimated with a logarithmic equation applied to the frequency–resistance relationship. Reliability for PhA@Fc and Fc was determined from two BIA assessments separated by at least 72 h.

**Results:**

A PhA type × dehydration interaction was found where both PhA@Fc and PhA@50 kHz significantly increased (*p* = 0.006; Δ: +1.41°); however, Holm post-hoc adjustments yielded no differences between them before (*p* = 0.336) or after (*p* = 0.053) the sauna intervention. Significant changes following dehydration were shown for Fc (*p* = 0.001; Δ: −1.97 kHz), R@50 kHz (*p* < 0.001; Δ: −6.6Ω), Xc@50 kHz (*p* = 0.002; Δ: −0.9Ω), and ICW (*p* = 0.035; Δ: +0.2 L), but not TBW or ECW (*p* > 0.05). Estimates of PhA@FC and Fc were shown to have excellent reliability (ICC > 0.90).

**Conclusions:**

Raw bioimpedance parameters and ICW are altered during acute, passive dehydration, whereas estimations of TBW and ECW may not be affected. Individualized values of PhA@Fc and Fc calculated from multi-frequency BIA are reliable measures; however, further investigation is needed to determine if they present unique responses to hypohydration.

